# Pharmacist-Led Interventions for Colorectal Cancer Prevention: A Systematic Review

**DOI:** 10.3390/curroncol33030177

**Published:** 2026-03-20

**Authors:** Zuzana Majsniarova, Daniela Minarikova, Peter Minarik, Tomas Fazekas, Jana Sremanakova

**Affiliations:** 1 Department of Organisation and Management in Pharmacy, Faculty of Pharmacy, Comenius University Bratislava, 832 32 Bratislava, Slovakia; majsniarova9@uniba.sk; 2 Institute of Experimental Endocrinology, Biomedical Research Center, Slovak Academy of Sciences, 845 05 Bratislava, Slovakia; peter.minarik@savba.sk; 3 Department of Physical Chemistry of Drugs, Faculty of Pharmacy, Comenius University Bratislava, 832 32 Bratislava, Slovakia; tomas.fazekas@uniba.sk; 4 Division of Nursing, Midwifery and Social Work, School of Health Sciences, The University of Manchester and Manchester Academic Health Science Centre, Manchester M13 9PL, UK; jana.sremanakova@manchester.ac.uk

**Keywords:** colorectal cancer, prevention, pharmacist, intervention

## Abstract

Colorectal cancer is the third most commonly diagnosed cancer and the second leading cause of cancer deaths worldwide, despite existing effective prevention strategies. Pharmacists are increasingly involved in various colorectal cancer prevention programs and are well-positioned to support these prevention efforts. In this systematic review, we investigate evidence regarding pharmacist-led interventions for colorectal cancer prevention by conducting a comprehensive search across the following electronic databases: MEDLINE, Embase, Web of Science, CINAHL, PubMed, and ClinicalTrials.gov. Studies were included only if they examined the role of pharmacists in early colorectal cancer detection and screening, lifestyle counseling to support prevention, and risk awareness and education. Risk of bias was assessed using the Pre–Post Quality Assessment developed by the National Institutes of Health. A total of 1405 studies were identified, of which four were included in the review. The included studies were quasi-experimental studies that mainly addressed screening awareness with fecal immunochemical test distribution, referrals, and counseling. The results of these studies showed an increase in screening participation, an improvement in knowledge of colorectal cancer risk factors and screening, and enhanced colorectal cancer referral rates. This systematic review highlights the potential of pharmacist-led interventions in enhancing colorectal cancer prevention efforts, but further well-designed studies are needed.

## Simple Summary

Colorectal cancer is a major health concern worldwide. Although preventive strategies such as early screening, lifestyle counseling, and risk awareness campaigns are affordable and could be effective in reducing its incidence, public awareness about colorectal cancer prevention and adherence to preventive measures are often poor and established public health strategies are generally insufficient. Pharmacists are increasingly contributing to various prevention programs and could support colorectal cancer prevention efforts. Hence, in this systematic review, we assessed pharmacist-led initiatives targeting colorectal cancer prevention. Although only a small number of studies were identified, the findings indicate that pharmacist-led activities can increase screening participation and improve knowledge of colorectal cancer prevention strategies. These results suggest that pharmacists could play a more substantial role in colorectal cancer prevention programs in the future.

## 1. Introduction

Colorectal cancer (CRC) is a major global health burden, ranking third in incidence and second in cancer-related deaths worldwide [1]. Globally, Hungary, Slovakia, Norway, the Netherlands, and Denmark had the highest age-standardized CRC incidence rates in 2020, ranging from 40.9 to 45.3 cases per 100,000 people [2]. Based on the most recent statistics, in Slovakia, 4219 new CRC cases were diagnosed in 2022, making it the most common malignancy in the country [3], with Slovakia having the third highest CRC mortality rate among men and the fifth highest among women in the EU [4].

CRC is one of the most frequently diagnosed cancers globally, although many cases can be prevented through better implementation of primary, secondary, and tertiary prevention strategies [5]. 

Recommendations for primary cancer prevention have been well-established by a large body of evidence [6,7]. Reducing body fat; limiting processed meat, sugar-sweetened beverages, and alcohol; avoiding smoking; increasing intake of fiber-rich foods, fruits, and vegetables; and engaging in physical activity are key lifestyle measures for reducing CRC risk [8]. To support adherence to cancer prevention recommendations, adequate public awareness is essential. However, available studies conducted worldwide [9] and in Slovakia [10] show that public awareness about these risk factors is often insufficient, with individuals having a tendency to underestimate the protective role of vegetables and fruits in their diet or to engage in physical activity [10].

The secondary prevention of CRC includes cost-effective screening programs aimed at reducing mortality through early disease detection and the removal of precancerous lesions. The best-known screening methods are the fecal occult blood test (FOBT), the fecal immunochemical test (FIT), sigmoidoscopy, and colonoscopy [11,12,13]. Although national CRC screening programs are available, significant disparities in their implementation exist [14], with low participation rates often recorded for national or European targets [15]. In Slovakia, an organized population-based CRC screening program has been accessible since 2019, aiming to improve screening rates [16]; however, participation in Slovakia still remains low. For instance, only <29% of invited individuals took part in CRC screening in 2023 [17]. The low participation in screening programs and the limited awareness of lifestyle-based CRC prevention strategies in Slovakia and other countries highlight the need for public health interventions and programs to increase public participation in screening, raise awareness of risk factors, and facilitate behavioral change to promote the adoption of preventive measures [18].

The role of pharmacists as healthcare professionals has changed significantly in recent years, with pharmaceutical care now going beyond the traditional dispensing of medicines to also focus on reducing the impact of diseases by providing screening and health promotion and education, monitoring public health threats, and promoting disease prevention and self-care [19]. Currently, pharmacists are primarily involved in the prevention and management of cardiovascular diseases [20,21], infectious diseases [22], and diabetes [23]. In the field of cancer care, pharmacists have contributed to tertiary prevention by guiding patients with cancer on how to manage treatment-related adverse events and complications [24]. They have also been increasingly engaged in cancer prevention activities such as distributing educational materials, encouraging screenings, participating in public health campaigns through the supply of self-test kits, and conducting structured referrals to other healthcare providers [9].

In a systematic review, Lindsey et al. summarized the existing studies focused on different cancer types and showed that it is feasible to recruit patients for education and screening interventions in community pharmacies. The included studies were mainly focused on prostate, breast, and cervical cancer, though some investigated colorectal cancer and were led by researchers and clinicians [25]. In the included studies on CRC, pharmacists’ roles were limited to the distribution of FIT/FOBT kits, without the provision of additional counseling on colorectal cancer prevention [25]. However, no pharmacist-led intervention studies focused on CRC were identified in the review.

Hence, little is known about the extent of pharmacists’ involvement in CRC prevention. Therefore, our aim in conducting this systematic review was to examine the latest evidence on pharmacist-led interventions in CRC prevention.

## 2. Materials and Methods

The protocol for this systematic review was submitted to PROSPERO (ID: CRD420250655305) and adheres to the PRISMA [26] (Preferred Reporting Items for Systematic Reviews and Meta-Analyses) guidelines and standards of Cochrane systematic reviews [26].

### 2.1. Search Strategy

Searches were conducted during the period between 6 April and 26 April 2025 in the following databases: MEDLINE via Ovid; MEDLINE via EBSCO; Embase via Ovid; Web of Science; the Cumulative Index to Nursing and Allied Health Literature (CINAHL); PubMed; and ClinicalTrials.gov. An updated search was conducted between 7 December 2025 and 4 January 2026. The search terms were aligned with MeSH vocabulary using truncation techniques and combined using Boolean operators. The complete search strategy is provided as an attachment in Supplementary Material S1.

### 2.2. Study Selection

The results of the database searches were uploaded to Rayyan [27], an online tool for systematic reviews. Duplicate records were identified and subsequently verified. Titles and abstracts were then independently reviewed by three reviewers (ZM, JS, and DM) according to predefined inclusion and exclusion criteria. Full-text articles were retrieved to assess eligibility, with screening conducted independently by ZM, JS, and DM. There were no limitations on publication date. Any disagreements that arose during the screening process were resolved through discussion between all authors. The results of the search and the study selection process are documented in the PRISMA flow diagram.

### 2.3. Inclusion Criteria

In this review, we included studies that met the following criteria: prospective studies, including both randomized and non-randomized controlled trials, as well as quasi-experimental studies targeting pharmacists delivering CRC prevention interventions to eligible adult participants. We included studies focused on CRC screening and early detection, lifestyle counseling supporting prevention, risk awareness and education targeting populations without CRC, populations at increased risk, and populations at risk of recurrence. We excluded studies in which CRC prevention constituted less than 25 percent of the study interventions or if the intervention was delivered by healthcare staff other than pharmacists. Also, patients receiving active treatment were excluded and only publications in English were considered.

### 2.4. Outcomes

The main outcomes assessed were CRC screening rates (such as FIT and FOBT uptake and colonoscopy) and reported measures of adherence to lifestyle interventions aimed at preventing CRC. Additional outcomes assessed were changes in knowledge and awareness of CRC and quality of life (QoL).

### 2.5. Data Extraction and Risk of Bias Assessment

Data from the included studies were extracted by one researcher (ZM) who collected information on baseline characteristics, including the first author, year of publication, full title, study location, funding sources, sample size, study design, duration, and follow-up period. Additional available details were gathered, such as age, sex, gender identity, health status, presence of comorbidities, and socioeconomic factors. Information was also gathered regarding the type of prevention and intervention assessed, intervention details, measured outcomes, and assessment methods and tools. Data extraction was independently reviewed by JS and DM. Any discrepancies were resolved through discussion among all authors. ZM, JS, DM, and TF conducted the risk of bias assessment using the Pre–Post Quality Assessment tool developed by the National Institutes of Health [28].

## 3. Results

### 3.1. Search Results

**Figure 1:**
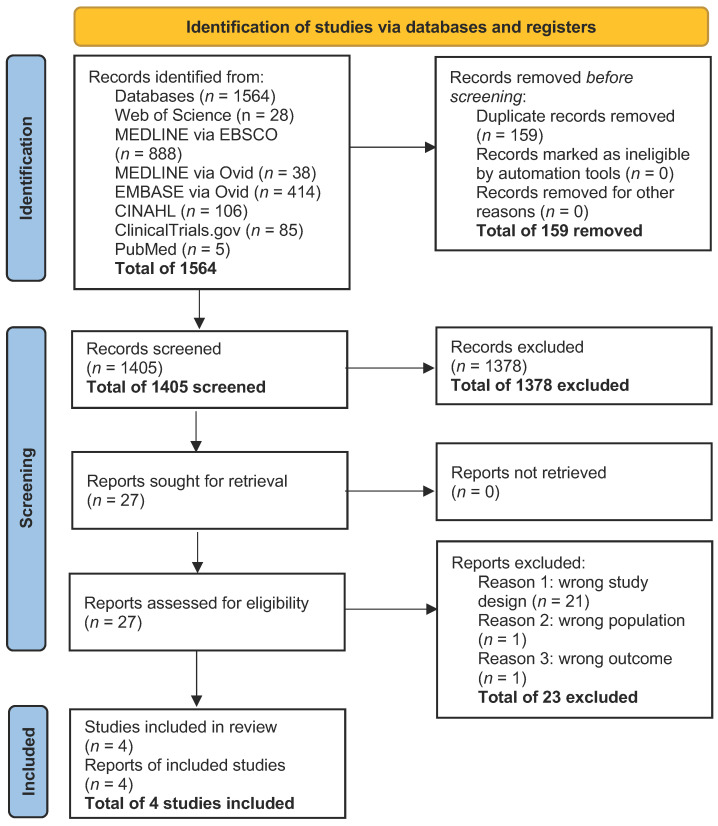
PRISMA diagram.

A total of four studies that fulfilled the inclusion criteria were included in this review [29,30,31,32]. Twenty-three studies were excluded due to having the wrong study design, wrong population, or wrong outcome; see the PRISMA diagram in Figure 1.

### 3.2. Study Characteristics

No randomized controlled trials were found; those included in the review were quasi-experimental before–after (pre–post) studies with no control group. Further information is provided in Table 1. The quality of the studies differed; two were rated as fair [30,31], one as poor [29], and one as good [32]. Details are presented in Table 2. The main issues across studies were missing or incomplete data, small sample sizes, and reliance on self-reported outcomes, while strengths included clear objectives, well-defined eligibility criteria, and valid outcome assessments.

### 3.3. Study Population Characteristics

The studies were conducted in the United Arab Emirates (*n* = 1), the United States (*n* = 2), and Australia (*n* = 1), with a total of 623 participants included. Participants’ ages ranged from 18 to 73 years. Jairoun et al. (2024), Holle et al. (2020), and Sriram et al. (2016) mainly included female participants [29,31,32], whereas Moore et al. (2023) primarily targeted males [30]. Jairoun et al. (2024) focused on individuals with chronic conditions such as type 2 diabetes and inflammatory bowel disease [29], while Moore et al. (2023) involved participants enrolled in chronic disease management programs, including those with cardiovascular diseases [30]. Holle et al. (2020) specifically targeted average-risk individuals without active disease [31], whereas Sriram et al. (2016) included patients presenting with bowel-related symptoms suggestive of carcinoma [32]. Details of participant characteristics are shown in Table 3.

### 3.4. Study Design Characteristics

The included studies were conducted in different pharmacy environments, such as independent and chain community pharmacies [29] and grocery-store-based pharmacy chains [30]. Participating pharmacies were chosen based on the provided pharmacy care services, such as chronic disease management [29,30], medication review, nutritional counseling [29], and disease prevention and medication management services [31]. In the study by Sriram et al. (2016), information about pharmacy care services was not included [32]. The locations of the pharmacies varied. Holle et al. (2020) involved pharmacies in underserved metropolitan areas [31], whereas Sriram et al. (2016) investigated twenty-one community pharmacies located in metropolitan and regional areas to reflect the diversity of socioeconomic sectors [32]. Jairoun et al. (2024) and Moore et al. (2023) did not state information about pharmacies’ locations [29,30]. More details about pharmacies and their services are shown in Table 4.

In order to prepare for the different prevention programs, pharmacists completed special training. In the study by Moore et al. (2023), the main topics of the training approaches were CRC risk factors, symptoms, prevention, screening opportunities, questionnaire administration, and counseling strategies [30]. In addition, in their study, each pharmacist reviewed the stool-based DNA test insurance coverage and effectiveness [30]. In the study by Sriram et al. (2016), pharmacy staff attended training sessions with the researcher to learn about the research protocol, recruitment, and study documentation [32]. More structured training was described in the study by Holle et al. (2020), where pharmacists completed a practice-based certificate program on communication skills for speaking with patients, different methods of CRC screening, risk factors for the disease, interventions, and motivational interviewing techniques [31]. The study by Jairoun et al. (2024) lacked information on pharmacists’ training [29].

In the reviewed studies, pharmacists used different types of educational resources and tests, including FITs combined with follow-up reminders [29,31], stool-based DNA tests [30], communication tools and educational materials [29], structured questionnaires, and CRC screening evaluation tools [30,32].

### 3.5. Study Findings

The findings from the reviewed studies highlight the range of pharmacist-led initiatives in CRC prevention, including risk assessment, knowledge improvement, screening awareness, and referral support. Across the included studies, several recurring patterns were evident, as shown in Table 4. Pharmacists were able to identify high-risk individuals and support the detection of previously undiagnosed CRC cases [30,32]. However, several barriers substantially limited the effectiveness of these studies. Moore et al. (2023) reported that only 17% of participants completed stool-based DNA testing, with cost, low perceived risk, and insufficient physician follow-up cited as major obstacles [30]. Holle et al. (2020) identified several barriers to study enrolment [31]. The primary barrier was a lack of interest in participation. Of the 312 individuals initially approached, only 16 provided consent to take part in the study. Among those who consented, 8 agreed to complete colorectal cancer (CRC) screening using the fecal immunochemical test (FIT) [31]. Sriram et al. (2016) found that pharmacist-led administration of the Jodi Lee test (validated self-administered questionnaire for detection, triage, and referral of bowel symptoms suggestive of carcinoma) improved referral rates compared with usual practice (38% vs. 20%), although some referrals were constrained by general practitioners’ awareness of symptoms or medication effects [32]. Jairoun et al. (2024) reported associations between CRC prevalence and age, inflammatory bowel disease, and type 2 diabetes mellitus, while aspirin use was linked to lower prevalence; however, FIT kit return rates were not documented, limiting interpretation [29].

## 4. Discussion

In this review, we aimed to evaluate the current evidence on pharmacists’ involvement in colorectal cancer prevention. To the best of the authors’ knowledge, this is the first systematic review focusing on interventions led by pharmacists as contributors to CRC prevention. The systematic review showed that no pharmacist-led randomized controlled trials targeting CRC prevention had been previously conducted, as only quasi-experimental studies without a control group were identified. The included studies primarily focused on increasing awareness and screening uptake. Although the methodological quality varied, the study findings collectively suggested that pharmacists could contribute to CRC prevention through several mechanisms: distribution of FIT or FOBT kits, provision of patient education, facilitation of referrals to physicians, and identification of individuals at increased risk. These roles are consistent with pharmacists’ broader integration into multidisciplinary healthcare teams, where they have already demonstrated value in screening programs for multiple cancer types [25].

### 4.1. Emerging Patterns

In this systematic review, we found that pharmacists could identify high-risk individuals and help in detecting previously undiagnosed CRC cases through organized programs. The reviewed studies found that pharmacist involvement could support CRC screening participation and test return rates [30,33], and training for pharmacists emerged as a critical determinant of success. Holle et al. (2020) further emphasized that pharmacists who completed certification programs in communication and motivational interviewing were better prepared to counsel patients [31], while Moore et al. (2023) similarly highlighted the importance of training in CRC risk factors, screening modalities, and counseling strategies [30]. These findings suggest that pharmacist readiness is important to facilitate CRC prevention efforts. However, some difficulties associated with pharmacists’ contributions to the CRC screening interventions need to be stated. In CRC screening programs, the establishment of well-designed tracking processes for stool-based tests is needed. Due to lack of time, pharmacists often could not take on responsibilities for referrals. Therefore, implementation of comprehensive tracking processes is important for future research to address the gap in follow-up colonoscopy management and improve the impact of CRC screening.

### 4.2. Comparison with Other Cancer Prevention Programs

Evidence from other cancer prevention initiatives supports the potential role of pharmacists in public health. For instance, pharmacist-led interventions have improved screening participation in breast cancer [34], prostate cancer [35], and cervical cancer [36]. Pharmacy-based breast cancer programs have improved knowledge and self-examination behaviors [34], while pharmacist-led prostate cancer initiatives have increased Prostate-Specific Antigen test acceptance [35]. Lindsey et al. (2015) and Miller et al. (2021) further demonstrated that pharmacists can enhance awareness and screening uptake across multiple cancer types [25], [37]. However, compared with CRC, these programs often involve less invasive testing, which may explain their higher participation rates. CRC screening requires stool samples or colonoscopy, procedures that are associated with stigma, discomfort, and logistical barriers. This distinction underscores the need for tailored approaches in CRC prevention, including targeted education to reduce stigma and structured reminder systems to improve test completion.

### 4.3. Lifestyle Interventions

Lifestyle counseling and interventions targeting behavioral change are key components of primary prevention strategies; however, none of the included studies incorporated such strategies. Jairoun et al. (2024) collected self-reported data on body mass index and did not assess other factors such as diet, physical activity, or behavioral change [17,29]. Studies that targeted lifestyle changes as an outcome did not meet the inclusion criteria of this review, as they were mostly led by researchers or healthcare professionals other than pharmacists, or they were focused on the prevention of cancer types other than CRC [38,39,40]. The breast cancer prevention study by Staynova et al. (2024) reported that pharmacists were already integrated into initiatives focused on lifestyle modification [40]. This would offer a potential opportunity for pharmacists to expand their role in CRC prevention activities [25,37]. Future research should therefore incorporate lifestyle adherence as a key outcome, alongside screening participation and knowledge improvement.

### 4.4. Strengths and Limitations

The strengths of this review are its rigorous approach and adherence to the PRISMA guidelines and standards of Cochrane systematic reviews [26]. However, the small sample sizes, reliance on self-reported outcomes, and incomplete data reporting reduce the reliability of the findings and are therefore the main study limitations [29,30,31,32]. The absence of control groups further limits causal inference. By including only studies focused on pharmacist-led interventions, we did not consider multi-stakeholder studies in which pharmacists collaborate with other stakeholders; such studies could potentially offer greater value and more effectively support colorectal cancer prevention efforts in practice. Also, our objective was to focus on the highest-level evidence from clinical trials; therefore, we limited inclusion to controlled trials and quasi-experimental studies. However, restricting eligibility only to these study designs may have reduced the total number of included studies and limited the complex results of this review. Only the English-language published literature was considered, and it was not possible to pool data and conduct a meta-analysis due to data heterogeneity and the small number of studies identified. These limitations highlight the need for larger, well-designed studies to establish best practices and evaluate the effectiveness of pharmacist-led CRC prevention initiatives.

### 4.5. Practical Implications

Despite these limitations, the findings have important practical implications. In countries such as Slovakia, with a long-lasting shortage of doctors and nurses, limited time to support prevention initiatives [41], and low CRC screening participation [17], pharmacists could be incorporated into national programs to strengthen prevention efforts. Potential roles include distributing FIT kits in community pharmacies, providing brief counseling and motivational support, and implementing reminder systems. Educational campaigns delivered in pharmacies could further raise awareness of CRC risk factors, screening opportunities, and the importance of prevention recommendations. Given pharmacists’ accessibility and trust within communities, their involvement could help bridge the gap between the availability of screening and actual participation.

Awareness and education about cancer prevention are an essential first step toward effective prevention, but their impact can be limited when implemented alone. Even when individuals understand CRC risk factors, adherence to healthy lifestyle recommendations can be constrained by environmental, economic, and social barriers. Factors such as the cost and affordability of healthy foods and the availability of exercise facilities and opportunities for physical activity within communities significantly affect whether people can follow these guidelines. These challenges highlight that multi-component strategies combining education with supportive policies, community infrastructure, and economic interventions are likely to have a greater impact on the practical adoption of cancer prevention behaviors.

### 4.6. Policy Recommendations

Pharmacists should be formally integrated into national CRC prevention and screening strategies, given their accessibility and their potential to improve screening uptake and support risk reduction. As highly accessible healthcare professionals, pharmacists are well positioned to deliver community-based interventions that complement existing primary care services and help reduce screening disparities.

To operationalize this integration, policymakers should establish clear reimbursement mechanisms for pharmacist-led services, including CRC risk assessment, fecal immunochemical test (FIT) kit distribution and counselling, lifestyle modification support, and structured referral pathways to primary care. In parallel, standardized training programmes, clinical protocols, and integrated digital risk assessment tools are necessary to ensure consistent, safe, and high-quality service delivery. Embedding community pharmacies within national public health campaigns, particularly those targeting underserved and hard-to-reach populations, could further enhance screening participation and improve early detection rates.

However, sustainable policy integration must be underpinned by robust evidence. At the research level, there is a clear need for high-quality, adequately powered randomized controlled trials, accompanied by economic evaluations, to determine the clinical and cost-effectiveness of pharmacist-led CRC interventions. Future studies should adopt standardized outcome measures, including screening uptake, referral rates, behavioral change, and long-term health impact. Strengthening the evidence base in this way will support informed policymaking and enable the sustainable implementation of pharmacy-based CRC prevention models within healthcare systems.

## 5. Conclusions

In summary, this systematic review provides an overview of studies focused on colorectal cancer prevention efforts led by pharmacists. The findings collectively demonstrate that pharmacists can contribute to CRC prevention through multiple roles. None of the included studies assessed lifestyle adherence as an outcome, despite strong evidence linking diet, physical activity, and weight management to reduced CRC risk, representing a significant gap in research and an opportunity for future studies to expand the scope of pharmacist-led interventions beyond screening. Future research should prioritize well-designed randomized controlled trials to establish best practices and determine the sustainability of pharmacist-led interventions. By building on strategies that have been successfully implemented in other cancer prevention programs, pharmacist-led CRC initiatives have the potential to become an integral component of comprehensive cancer prevention strategies.

## Figures and Tables

**Table 1:** Study characteristics.

Study Reference	Full Title	Study Location	Funding Sources	Number of Participants	Study Design	Study Duration	Follow-Up Period
Jairoun et al. (2024) [29]	Community pharmacist-led point-of-care CRC screening program: Early detection of CRC in high-risk patients	United Arab Emirates	No funding reported	401	Prospective pre–post	10 months	No
Moore et al. (2023) [30]	Assessing the effects of pharmacist-provided education on CRC screening and access to a stool-based DNA test	United States of America	American Pharmacists Association Foundation (incentive grant)	42	Prospective Pre–post	4 months	No
Holle et al. (2020) [31]	Pharmacist-led intervention in a CRC screening initiative	United States of America	UCHC-UConn Storrs Seed Grant Funding in Cancer Control program; FIT kits were donated by Polymedco (Seattle, WA)	16	Prospective pre–post	Not clearly specified	Up to 45 days (two telephone follow-ups), follow-up survey of CRC knowledge and program satisfaction
Sriram et al. (2016) [32]	A model for assessment and referral of clients with bowel symptoms in community pharmacies	Australia	Jodi Lee Foundation PhD scholarship	164 (84 Usual practice; 80 JLT)	Prospective pre–post	11 months	4+ weeks post-visit, telephone follow-up

FIT—fecal immunochemical test; JLT—Jodi Lee test (validated self-administered questionnaire for detection, triage, and referral of bowel symptoms suggestive of carcinoma); CRC—colorectal cancer.

**Table 2:** Risk of bias assessment.

Question	Jairoun et al. (2024) [29]	Moore et al. (2023) [30]	Holle et al. (2020) [31]	Sriram et al. (2016) [32]
1. Was the study question or objective clearly stated?	yes	yes	yes	yes
2. Were eligibility/selection criteria for the study population prespecified and clearly described?	yes	yes	yes	yes
3. Were the participants in the study representative of those who would be eligible for the test/service/intervention in the general or clinical population of interest?	yes	no	no	yes
4. Were all eligible participants that met the prespecified entry criteria enrolled?	CD	no	no	CD
5. Was the sample size sufficiently large to provide confidence in the findings?	no	no	no	yes
6. Was the test/service/intervention clearly described and delivered consistently across the study population?	CD	yes	yes	yes
7. Were the outcome measures prespecified, clearly defined, valid, reliable, and assessed consistently across all study participants?	no	yes	yes	yes
8. Were the people assessing the outcomes blinded to the participants’ exposures/interventions?	no	no	no	no
9. Was the loss to follow-up after baseline 20% or less? Were those lost to follow-up accounted for in the analysis?	NA	CD	no	yes
10. Did the statistical methods examine changes in outcome measures from before to after the intervention? Were statistical tests done that provided *p* values for the pre-to-post changes?	no	yes	yes	yes
11. Were outcome measures of interest taken multiple times before the intervention and multiple times after the intervention (i.e., did they use an interrupted time-series design)?	no	no	no	no
12. If the intervention was conducted at a group level (e.g., a whole hospital, a community, etc.) did the statistical analysis take into account the use of individual-level data to determine effects at the group level?	no	NA	NA	CD
**Quality rating**	**poor**	**fair**	**fair**	**good**

CD—cannot determine; NA—non-applicable.

**Table 3:** Study population characteristics.

Study Reference	Age	Sex	Health Status	Comorbidities	Socioeconomic Factors
Jairoun et al. (2024) [29]	Mean: 66.6 ± 11.3	Male: 39.9%, Female: 60.1%	Obesity and overweight in those with high risk of CRC	Type 2 diabetes (85%), inflammatory bowel disease (10.5%)	Not stated
Moore et al. (2023) [30]	Median: 59 (range: 52–62.5)	Male: 51.2%, Female: 48.8%	Chronic disease management program (diabetes and cardiovascular diseases)	Type 1/2 diabetes, hypertension, dyslipidemia, ASCVD	Most had ≤college education
Holle et al. (2020) [31]	45+	Female: 56.2%, Male: 43.8%	No active disease in those with risk of CRC according to the National Comprehensive Cancer Network Clinical Practice Guidelines in Oncology	Not reported	Socioeconomically disadvantaged population: 68.75% unemployed and 62.5% without college education
Sriram et al. (2016) [32]	18+	Female: 71%, Male: 29%	Participants with bowel symptoms suggestive of carcinoma	Not reported	Diverse socioeconomic population

CRC—colorectal cancer; ASCVD—atherosclerotic cardiovascular disease.

**Table 4:** Study design characteristics.

Study Reference	Study Purpose	Outcomes Measured	Assessment Tools	Participant Resources	Training for Pharmacists	Details on Pharmacies	Study Findings
Jairoun et al. (2024) [29]	FOBT kit distribution, identification of individuals at increased risk	FIT completion and CRC prevalence	FIT, demographic and baseline characteristic questionnaire, and reminder phone calls or text messages twice a month	Face-to-face counseling, communication materials regarding appropriate prevention strategies and risk of CRC	Not reported	Six chain and independent community pharmacies	Statistically significant association between increased prevalence of CRC and age, inflammatory bowel disease, and diabetes mellitus 2. Decreased prevalence of CRC among aspirin users. No data on FIT kit return rates.
Moore et al. (2023) [30]	Provision of patient education, FOBT kit distribution	Knowledge score, perception of risk of developing colon cancer, and test completion	16-item pre/post questionnaire targeting demographics, knowledge about CRC, screening intentions and barriers, stool-based DNA test, GP referrals, and CRC status evaluation tool	Questionnaire and verbal dispensation and written materials about CRC risk factors, symptoms, and prevention	Education offered by the primary researcher about questionnaire administration, risk factors for CRC, screening initiation, prevention, stool-based DNA test insurance coverage, and effectiveness	Regional grocery store chain of pharmacies involved in the pharmacist-led chronic disease state management program	Significant increase in knowledge scores. The three most common reported barriers to completing CRC screening were identified (cost of screening, not being concerned with colon cancer, and lack of follow-up from a physician). A total of 42 participants completed the questionnaires and 17% of participants underwent screening during the study.
Holle et al. (2020) [31]	FIT distribution, provision of patient education	FIT completion and CRC knowledge improvement (CRC risk factors, tests, symptoms, screening options)	FIT, CRC risk score calculation, knowledge pre/post questionnaire, and two follow-up call reminders	Written instructions on sample collection and return and face-to-face counseling on proper use of FIT and risk reduction mechanisms	Practice-based certificate program—online didactic learning + in-person training about CRC screening methods, statistics, risk factors, and communication skills for speaking with patients and motivational techniques	UConn Health Colon Cancer Prevention Program and UConn School of Pharmacy, in collaboration with major independent chain pharmacies in underserved metropolitan areas	After approaching 312 people, 16 participants enrolled in the study, with 8 participants agreeing to complete FIT. There was one positive FIT, but the follow-up colonoscopy for that patient was negative. In the baseline CRC knowledge survey, 16 participants answered an average of 2.6 questions incorrectly. Follow-up (*n* = 4) results were similar.
Sriram et al. (2016) [32]	Facilitation of referrals to physicians	GP referral rate, GP visit rate, and diagnosis outcome	JLT questionnaire compared vs. usual practice, post-trial staff survey conducted on the usability of JLT, and recommendations for future	No resources reported; consultations with pharmacists about symptoms, medications used, and referrals were offered	Training session on the research protocol and instructions on the recruitment and study documentation	21 community pharmacies	A total of 80 participants were observed for 20 weeks; 50 of them (63%) were identified by the JLT for GP referral, but pharmacists confirmed only 30 (38%), often due to GP awareness of symptoms or medication side effects. Intervention Phase versus the Usual Practice Phase referral rates were 38% vs. 20%.

CRC—colorectal cancer; FIT—fecal immunochemical test; GP—general practitioner; JLT—Jodi Lee Test (validated self-administered questionnaire for detection, triage, and referral of bowel symptoms suggestive of carcinoma).

## Data Availability

No new data were created in this study.

## Supplementary Materials

The following supporting information can be downloaded at: https://www.mdpi.com/article/10.3390/curroncol33030177/s1, Supplementary Material S1: Search Strategy.
