# Supplementary material for: Pharmacist-Led Interventions for Colorectal Cancer Prevention: A Systematic Review
Source: Curr Oncol. 2026 Mar 20;33(3):177. doi: 10.3390/curroncol33030177 (PMC13025529; doi:10.3390/curroncol33030177)
Supplement: Supplementary file 1 [file curroncol-33-00177-s001.zip › curroncol-4141175-supplementary.pdf]

Search 04/01/2026 – MAIN SEARCH STRATEGY

1. Search Blocks by Concept

Block A: Colorectal Neoplasms or Bowel Cancer

1. exp Colorectal Neoplasms/
2. (colorectal cancer or colon cancer or rectal cancer or bowel cancer or CRC).tw,kf.
3. 1 OR 2

Block B: Pharmacist-Led or Pharmacy-Based Interventions

4. exp Pharmacists/
5. (pharmacist-led or pharmacist delivered or pharmacy-based or clinical pharmacist or community pharmacist).tw,kf.
6. 4 OR 5

Block C: Interventions / Services / Education / Screening

7. exp Health Education/ OR exp Patient Education as Topic/ OR exp Health Services/
8. (intervention* or program* or strategy* or service* or screening or education or support or management or initiative*).tw,kf.
9. 7 OR 8

---

2. Combine Concepts

10. 3 AND 6 AND 9
